# Bioinspired soft robots for deep-sea exploration

**DOI:** 10.1038/s41467-023-42882-3

**Published:** 2023-11-04

**Authors:** Guorui Li, Tuck-Whye Wong, Benjamin Shih, Chunyu Guo, Luwen Wang, Jiaqi Liu, Tao Wang, Xiaobo Liu, Jiayao Yan, Baosheng Wu, Fajun Yu, Yunsai Chen, Yiming Liang, Yaoting Xue, Chengjun Wang, Shunping He, Li Wen, Michael T. Tolley, A-Man Zhang, Cecilia Laschi, Tiefeng Li

**Affiliations:** 1 Qingdao Innovation and Development Base, Harbin Engineering University, Qingdao, China; 2 Science and Technology on Underwater Vehicle Technology Laboratory, Harbin Engineering University, Harbin, China; 3 College of Shipbuilding Engineering, Harbin Engineering University, Harbin, China; 4 Center for X-Mechanics, Zhejiang University, Hangzhou, China; 5 Department of Biomedical Engineering and Health Sciences, Universiti Teknologi Malaysia, Skudai, Malaysia; 6 School of Engineering and Applied Sciences, Harvard University, Cambridge, MA USA; 7 School of Information and Electrical Engineering, Hangzhou City University, Hangzhou, China; 8 School of Mechanical Engineering and Automation, Beihang University, Beijing, China; 9 Department of Mechanical and Aerospace Engineering, University of California, San Diego, MA USA; 10 School of Ecology and Environment, Northwestern Polytechnical University, Xi’an, China; 11 Zhejiang Lab, Hangzhou, China; 12 Institute of Deep-Sea Science and Engineering, Chinese Academy of Sciences, Sanya, China; 13 Department of Mechanical Engineering, National University of Singapore, Singapore, Singapore; 14 The BioRobotics Institute, Scuola Superiore Sant’Anna, Pisa, Italy

**Keywords:** Mechanical engineering, Polymers

## Abstract

The deep ocean, Earth’s untouched expanse, presents immense challenges for exploration due to its extreme pressure, temperature, and darkness. Unlike traditional marine robots that require specialized metallic vessels for protection, deep-sea species thrive without such cumbersome pressure-resistant designs. Their pressure-adaptive forms, unique propulsion methods, and advanced senses have inspired innovation in designing lightweight, compact soft machines. This perspective addresses challenges, recent strides, and design strategies for bioinspired deep-sea soft robots. Drawing from abyssal life, it explores the actuation, sensing, power, and pressure resilience of multifunctional deep-sea soft robots, offering game-changing solutions for profound exploration and operation in harsh conditions.

## Abstract (web summary)

High pressure and low temperature are the greatest challenges faced by scientists to explore deep oceans, which remain largely unknow to us today. Li et al. review these challenges and give insight into designing soft robots, inspired by deep-sea creatures, that enable resilient operations in harsh conditions.

## Introduction

The exploration of Earth’s oceans presents a highly significant endeavor, holding the potential to unravel oceanic mysteries^1^ and unveil concealed marine biodiversity^2–4^, energy resources^5–8^, and mineral reserves^9,10^. Despite recent advances in exploring the ocean’s shallow layers^11–13^, the profound depths of the deep sea present daunting challenges marked by extreme hydrostatic pressure, chilling temperatures, and perpetual darkness^14–16^. Defined as ocean regions between 1000 and 11,000 m in depth, the deep sea remains 95% uncharted. This vast unexplored domain has spurred the development of deep-sea robotic technology^14,17–22^, and empowering humanity to accelerate the discovery of these untrodden frontiers.

**Fig. 1:**
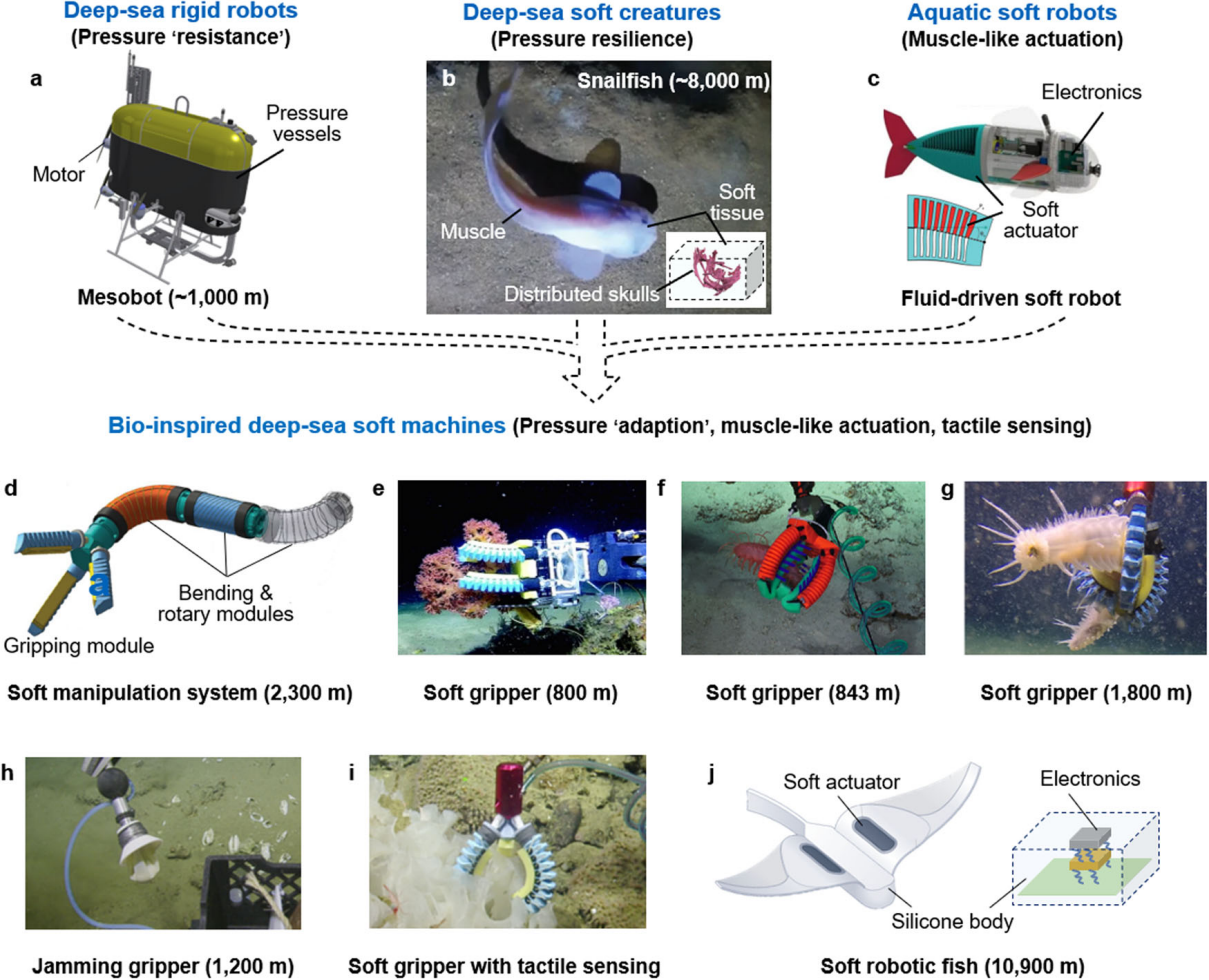
Evolutionary design of deep-sea soft machines. **a** Pressure-resistant designs such as pressure vessels are widely used to protect the mechatronics in deep-sea rigid robots^26^. Reproduced with permission from ref. ^26^, copyright 2021, The American Association for the Advancement of Science. **b** Deep-sea snailfish possess remarkable adaptability and survivability under harsh deep-sea conditions. Their distributed skulls and muscle actuation provide abundant bioinspiration for designing multifunctional deep-sea soft machines^35^. Reproduced with permission under CC BY 4.0 license from ref. ^35^. **c** Soft robots are mainly composed of elastic materials and can generate muscle-like actuation for various tasks such as swimming^52^. Reproduced with permission from ref. ^52^, copyright 2018, The American Association for the Advancement of Science. Previous demonstrations of deep-sea soft robots include: **d** A dexterous soft manipulator that integrates bending, rotary, and grasping units for deep-sea manipulation^60^; Reproduced with permission under CC BY 4.0 license from ref. ^60^. **e**–**g** Soft robotic grippers perform delicate collection of fragile deep-sea specimens at depths of 800 m^61^, 843 m^62^ and 1800 m^60^, respectively; **e**–**g** Reproduced with permission under CC BY 4.0 license from refs. ^60–62^. **h** A soft jamming gripper was field-tested at a depth of 1200 m^63^; Reproduced with permission from ref. ^63^, copyright 2017, Mary Ann Liebert, Inc. **i** A deep-sea soft gripper was integrated with a waveguide-based tactile sensor^64^; Reproduced with permission from ref. ^64^, copyright 2018, IEEE. **j** A bioinspired soft robot that is able to operate at a depth of 10,900 m^66,67^. Reproduced with permission from ref. ^67^, copyright 2021, Springer Nature.

Deep-sea vehicles and mobile robots are pivotal for various oceanic missions^23–26^ but are often encased in ‘pressure-resistant’ designs that encompass metallic pressure enclosures^26–28^ and pressure-compensation systems. These protective measures safeguard the embedded electronics and mechatronic systems from the relentless pressures of the deep sea^26^ (Fig. 1a). However, these hard machines face limitations, including increased weight, risk of structural fatigue failure, and restricted adaptability when dealing with delicate deep-sea specimens^29–32^. The incident involving the Nereus HROV, a prominent deep-sea vehicle, exemplifies these challenges when its onboard pressure vessel imploded at a depth of 9900 m^30^. Consequently, researchers have turned to deep-sea organisms for inspiration, seeking innovative solutions to enhance the resilience and agility of deep-sea exploration technology.

In contrast, deep-sea creatures thrive in extreme depths^33–37^, displaying remarkable pressure resilience, mobility, and sensory acumen (Fig. 1b). Soft robots, inspired by these remarkable organisms, replicate muscle-like actuation and theoretically offer unlimited degrees of freedom^38–45^. Their fully soft-bodied structures, composed of pliable materials and highly deformable components, enable gentle interactions with fragile specimens^45–49^ and adaptability in unstructured environments^50–54^ (Fig. 1c). Additionally, the concept of morphological intelligence, observed in deep-sea creatures like octopuses, has influenced the design of soft robots endowed with mechanical intelligence. This approach simplifies control, streamlines algorithms, and reduces the reliance on complex control systems and computing hardware, which may be particularly valuable in harsh deep-sea conditions^55–57^. Innovations such as soft actuators inspired by muscular hydrostats, fluidic-driven soft actuators inspired by hydrostatic skeletons^58–65^, and dielectric elastomer-driven soft robots with decentralized electronics^66,67^ exemplify the pivotal role of bioinspiration in the development of soft machines for deep-sea exploration (Fig. 1d–j). This perspective explores the principles of designing deep-sea soft machines, from bioinspired insights to their applications in actuation, perception, and pressure resilience. It also highlights recent achievements in delicate manipulation and untethered locomotion, and outlines future directions in this field, including enhancing locomotor and sensing capabilities, and pursuing sustainable development for deep-sea soft machines.

### Bioinspired deep-sea soft machines

The fields of ‘aquatic soft robotics’ and ‘deep-sea exploration’ have both seen rapid progress in recent decades. Thanks to the abundance of inspirations from deep-sea creatures, deep-sea soft robots are likely to demonstrate limitless potential for deep-sea tasks such as delicate manipulation and non-destructive exploration. However, incorporating advances from these two fields remains a daunting technical challenge, due in part to a lack of established design principles and the complexities of combining key techniques. Therefore, more collaborations among roboticists, biologists, material scientists, and engineers are needed to construct bioinspired soft robots capable of exploring the vast uncharted oceans. Approaches for actuation, sensing, and mechanical design must be redefined to address the challenges posed by these harsh conditions. In essence, comprehending the impact of depth on these tasks constitutes a fundamental question. Here, we refine the major existing questions and aim to provide solutions for designing deep-sea soft robots from a bioinspired viewpoint. We also outline representative applications in this area.

### Designing principles for soft actuation in the deep ocean

#### Bioinspired deep-sea hydraulic soft actuation

**Fig. 2:**
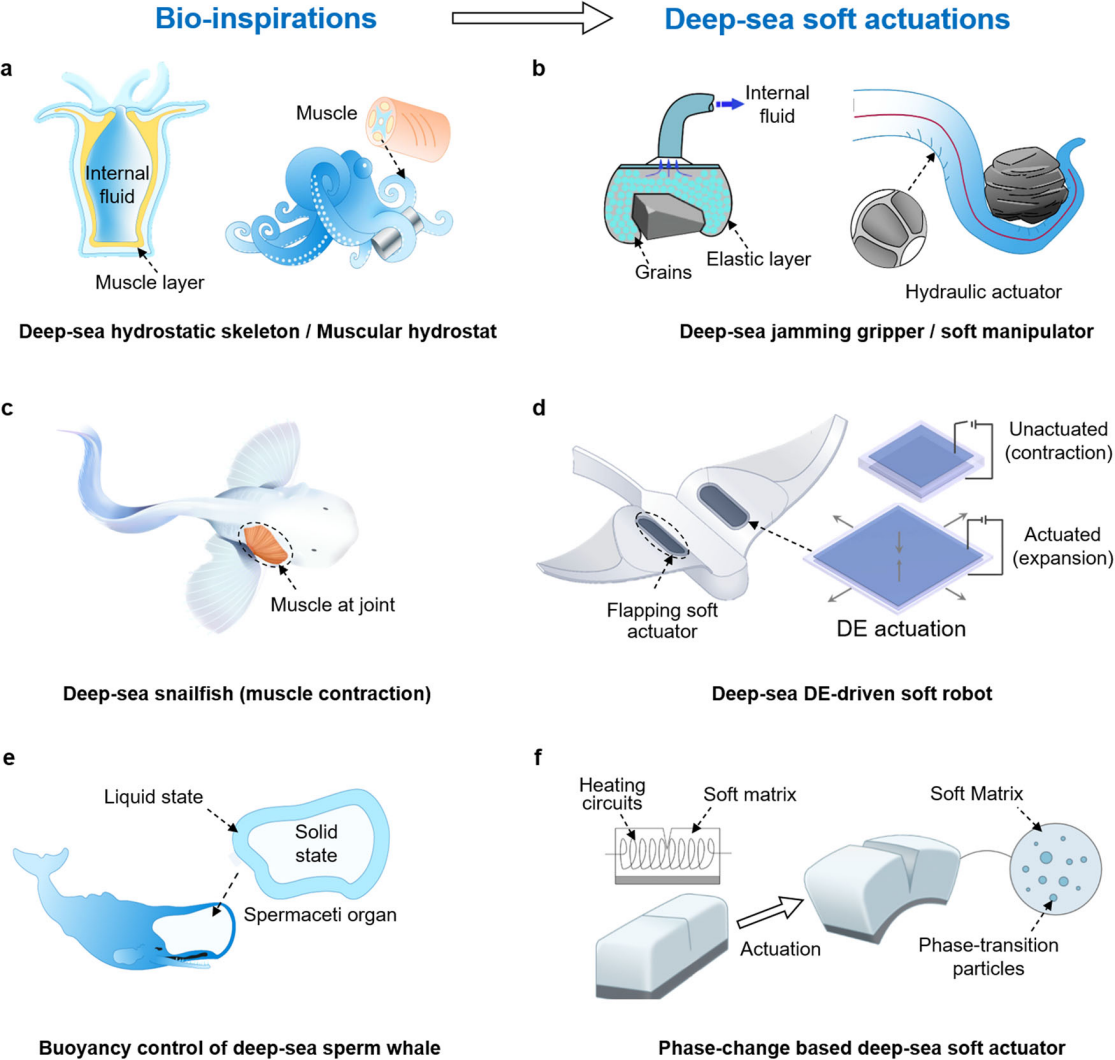
Bioinspired deep-sea soft actuation. **a** Deep-sea invertebrate (e.g., sea anemones, octopuses) achieve actuation and various movements (e.g., manipulation) using their hydrostatic skeletons and muscular hydrostats^68^, which inspire the design of (**b**) a hydraulic jamming gripper for implementing universal deep-sea grasping^63^ and a dexterous soft manipulator. **c** Deep-sea vertebrates utilize the contraction and expansion of their muscles attached to the skeleton to generate a driving force for locomotion^66^. Reproduced with permission from ref. ^66^, copyright 2021, Springer Nature. **d** When a voltage is applied on a DEA membrane, the induced electrostatic force leads to a decrease in its thickness and an expansion in its area, which has been utilized in the flapping actuation of deep-sea soft robot^67^. Reproduced with permission from ref. ^67^, copyright 2021, Springer Nature. **e** The sperm whale achieves buoyancy control by regulating the liquid-solid phase change of its spermaceti organ^72^. **f** Inspired by the phase-change mechanism of the spermaceti organ, soft actuators integrating phase-change materials have the potential to be used for lightweight actuation under pressure conditions^73^. Reproduced with permission under CC BY 4.0 license from ref. ^73^.

Deep-sea invertebrates utilize hydrostatic skeletons and muscular hydrostats to support their soft bodies, achieve actuation, and enable locomotion under extreme pressures^33,37,68^. A hydrostatic skeleton typically consists of a fluid-filled cavity (coelom) surrounded by muscle layers, which act against the internal fluidic pressure to generate movement^58,59^. For example, sea anemones regulate their body shape by actively manipulating internal water, functioning like hydraulic pumps (Fig. 2a). By contrast, a muscular hydrostat is a structure composed of densely arranged muscles, providing skeletal support and generating the forces necessary for movement and deformation. Deep-sea octopuses, similar to an elephant’s trunk, execute dexterous manipulations with their entirely soft arms (Fig. 2a).

Largely inspired by the hydrostatic skeletons of deep-sea invertebrates, hydraulic soft actuators have been developed. These actuators consist of a flexible chamber filled with water that acts as both pressure compensation and actuation fluids^61^. Due to the pressure balance between its internal chamber and the surrounding open water, this type of soft actuator is inherently suitable for operation under high hydrostatic pressure conditions. When the internal fluid (water) is pumped into or out of the the mechanically programmed chamber, multi-degree-of-freedom (multi-DOF) motions including extending, bending, and wrestling can be executed^47,60^.

Additionally, a universal jamming gripper serves as another option for deep-sea soft manipulation. This gripper consists of a flexible balloon filled with granular particles and fluid. When the internal fluid is pumped out, the grains (granular particles) jam together, causing deformation in the balloon generating grasping force^63^ (Fig. 2b). This strategy enables several soft manipulators to delicately envelope and manipulate fragile deep-sea specimens^60–64^ (Fig. 2b). However, these soft actuators still require hydraulic pumps and pressure-regulating components. The bulky mechatronic components typically used for these functions pose significant challenges in the design and system integration of untethered deep-sea soft robots based on hydraulics.

#### Muscle-like deep-sea soft actuation

Deep-sea vertebrates swim by exerting force against the surrounding water. The periodic contraction and relaxation of the muscles, attached to their supporting skeletons, generate forward thrust and control the swim direction^66^ (Fig. 2c). Artificial muscles such as dielectric elastomer actuators (DEAs) have the potential to provide a similar function in soft actuation^69^. When a voltage is applied on a DE membrane, the induced electrostatic force deforms the membrane, causing a decrease in its thickness and an increase in its area^67,70^ (Fig. 2d). In contrast to the aforementioned hydraulic soft actuators, DEAs are easily untethered powered and controlled, and have been widely used in aquatic soft robots^51,66^. DEAs stand out among stimuli-responsive actuators due to their large actuation, rapid response, and low power consumption. These features make them particularly suitable for deep-sea soft machines, especially when compared to thermally responsive actuators for periodic soft actuation task. However, it should be noted that a decrease in this voltage-induced strain has been observed under harsh deep-sea conditions^66^. Dynamic mechanical analysis (DMA) and in-situ tensile tests have confirmed that this decrease is due to the increased hardness of the soft material under low temperatures and high pressures. To address this drawback, an optimized DEA material with a lower glass-transition temperature (*T*_g_) has been employed to enhance its voltage-induced actuation in the deep sea^71^.

#### Thermal based phase-transition material for deep-sea actuation

Without the need of internal air bladder, sperm whales can attain neutral buoyancy at various depths by controlling the temperature and phase transition (liquid-solid) of their spermaceti organ^72^ (Fig. 2e). This serves as a potential solution for compact buoyancy regulation for deep-sea soft robots. By integrating distributed ethanol micro-bubbles into silicone materials, a phase-change soft actuator achieves a strain up to 900%^73^ (Fig. 2f). However, the high pressure in the deep sea remains a significant obstacle for achieving the phase change from liquid to gas^74^. In this regard, a wax-filled chamber integrated with heating circuits can achieve a volume change of 10–15%, coupled with the phase-change from solid to liquid, even at pressures up to 200 MPa^75^. This actuator has also been used to adjust buoyancy at the depth of 3223 m. Future efforts may focus on engineering soft materials capable of phase transition with better energy efficiency and faster response speeds.

### Designing principles for soft machine pressure resilience in the deep ocean

**Fig. 3:**
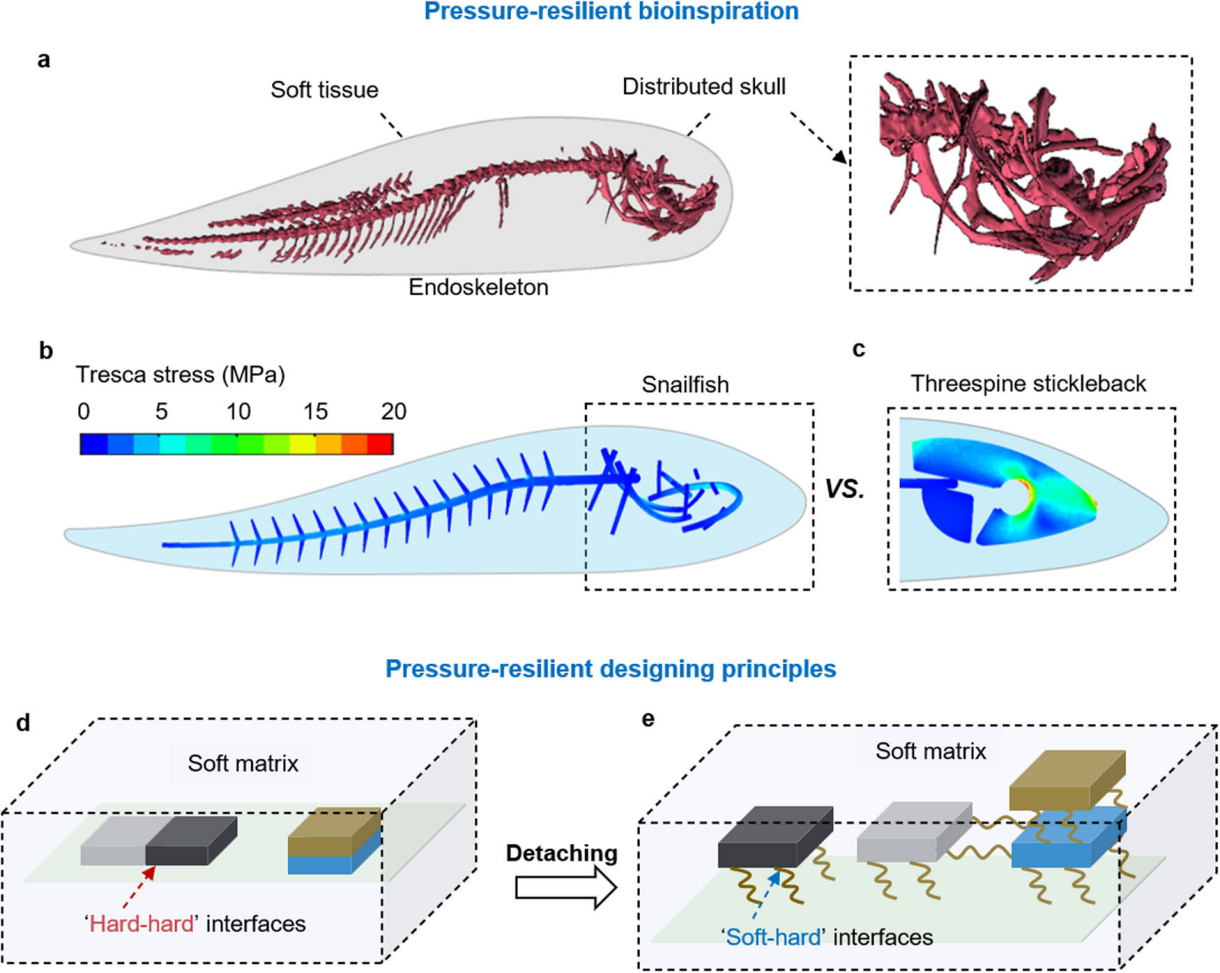
Pressure-resilient design of bioinspired deep-sea soft robots. **a** As a deep-sea animal with an endoskeleton, the snailfish possesses a low modulus internal skeleton and a distributed skull in its soft tissue. **b**, **c** Despite experiencing the same value of hydrostatic pressure, the maximum shear stress on the distributed skulls of the snailfish is significantly lower than that of the stickleback fish from shallow water. **d**, **e** The distributed skulls of snailfish serve as inspiration for the design of detaching electronics, which enhances their pressure resilience in the deep sea^66^. Reproduced with permission from ref. ^66^, copyright 2021, Springer Nature.

Deep-sea creatures have evolved special adaptations that allow them to survive the extreme pressure. For those without rigid bones (e.g., sea anemones), a fully soft-bodied structure, completely filled with internal fluid, enables both inherent pressure resilience and actuation^33^. This insight could be valuable for designing a fully soft hydraulically-driven deep-sea robot. In a recent surprising discovery, hadal snailfish was found to inhabit ocean depths below 8000 m^35,76^. Unlike the bulky and fully enclosed skulls of three-spine stickleback fish that inhabit shallow waters^77^, the skulls of hadal snailfish are partially enclosed and appear as smaller pieces scattered throughout soft tissue, which helps to mitigate the shear stress induced by extreme hydrostatic pressure (Fig. 3a). Finite element analysis (FEA) reveals significant differences in shear stress distribution between shallow-water stickleback fish^77^ and the hadal snailfish^35^. Under the same hydrostatic pressure (i.e., 110 MPa), the maximum shear stress on the snailfish’s skulls to be approximately 7 MPa, or less than half of the shear stress that is acting on the stickleback fish (approximately 18 MPa) (Fig. 3b, c).

The morphology of the hadal snailfish (e.g., a low-modulus skeleton, distributed skulls) has served as inspiration for the ‘pressure-resilient’ design of a deep-sea soft robot capable of surviving intense hydrostatic pressure^66^. In this design, the onboard electronics are wire-connected or separately distributed onto small PCBs, which are then encapsulated within a soft matrix in a decentralized manner (Fig. 3d, e). FEA results shows that the shear stress between the decentralized electronics is effectively mitigated under a hydrostatic pressure of 110 MPa, compared to the centralized design where the electronic were densely packed. Furthermore, a series of experiments are conducted to validate pressure resilience of decentralized electronics embedded in a soft matrix^66^.

This ‘decentralized’ design approach for mitigating the shear stress distribution of encapsulated electronics has been refined as the strategy of detaching, distancing, and eliminating voids. Specifically, ‘detaching’ means eliminating ‘hard-hard’ interfaces by removing the direct contacts among the rigid components with distinct mechanical contrast. ‘Distancing’ refers to increasing the spaces between neighboring electronics on PCBs. To ensure proper functionality under extreme pressures, the use of electronics without internal voids is a must, for instance, ceramic capacitor is an ideal alternative to electrolytic ones. Future directions may involve flexible electronics and soft circuits (e.g., liquid metal), which also offer promising solutions for integrated power, control, and sensing of fully soft-bodied intelligent machines, owing to their inherent pressure resilience in the deep sea^78,79^.

### Designing principles for soft machine sensing in the deep ocean

#### Tactile sensing

**Fig. 4:**
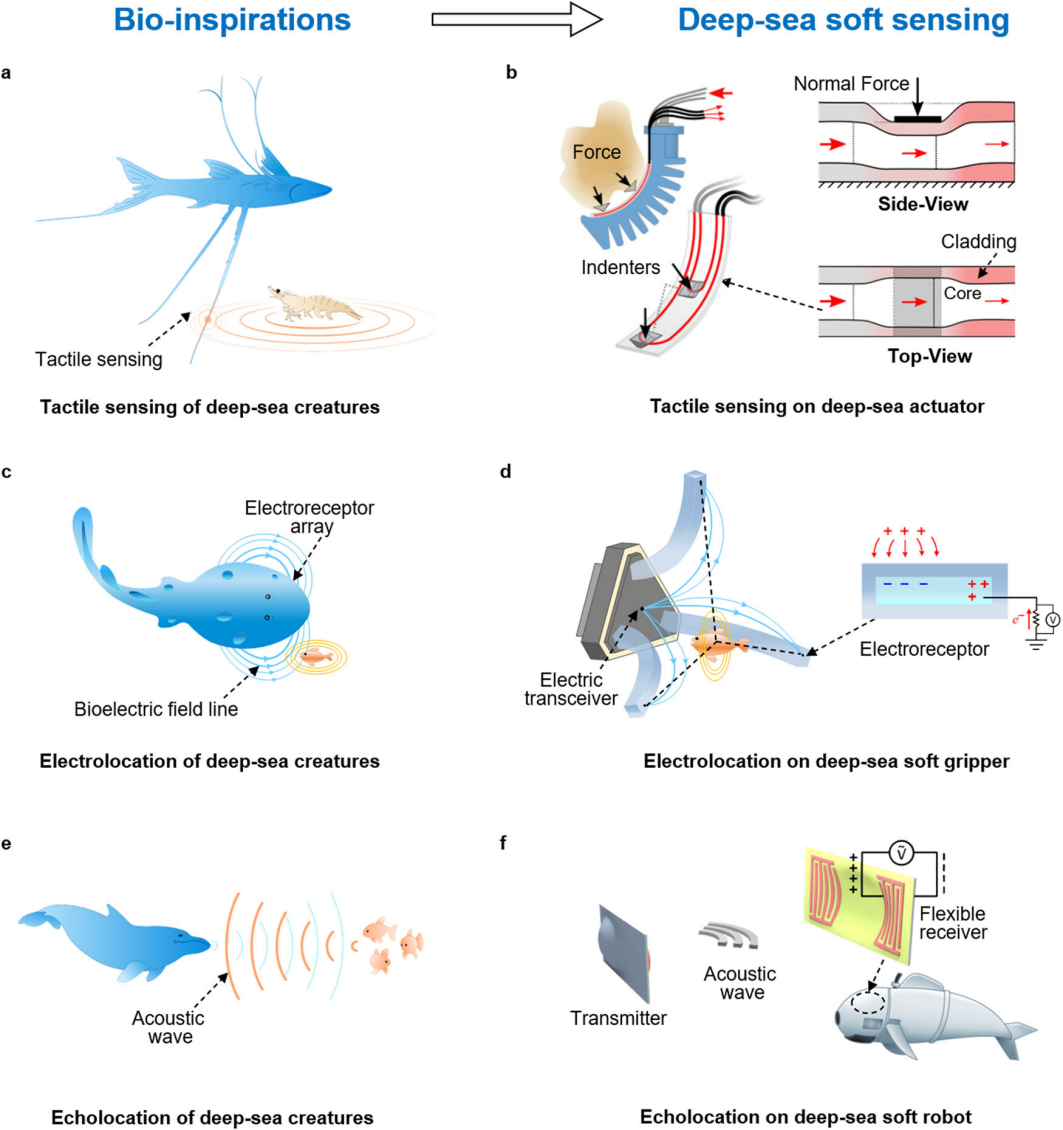
Bioinspired deep-sea soft sensing. **a** Many deep-sea creatures such as tripod fish have evolved long appendages to enhance their tactile sensitivity^80^, which has inspired the design of (**b**) waveguide deep-sea tactile sensor^64^. Reproduced with permission from ref. ^64^, copyright 2018, IEEE. **c** Benthobatis moresbyi have special organs to emit electric field and electroreceptor arrays to detect its surrounding objects^84^. **d** The electric sensory organs of deep-sea fish inspire the potential design and integration of flexible electroreceptor array for electrolocation of soft machines^85^. Reproduced with permission under CC BY-NC 4.0 license from ref. ^85^. **e** Cetaceans detect surrounding objects using echolocation in darkness^86^, inspiring (**f**) a flexible acoustic transceiver composed of piezoelectric electrodes attached to a flexible substrate for potential distance communication and sensing of deep-sea soft robot^52,87^. Reproduced with permission under CC BY 4.0 license from ref. ^87^.

Deep-sea creatures have impressively evolved with a variety of sensory modalities to intensify situational awareness for dealing with the complicated situations in the deep sea. To deal with the total darkness, some deep-sea fish (e.g., lizardfish, tripod fish), have developed long appendages, abundant spines, and other unique organs to enhance their tactile sensitivity^80^ (Fig. 4a). Sea robins walk on the ocean floor and use whiskers consisting of bony plates along their bodies and branched barbels to navigate their terrain^81^. Besides, fish can detect the presence of object in the surrounding water through the lateral line system of mechanosensors, facilitated by hydrodynamics wakes created by the object. Although there is no physical touching between the fish and object in this situation, it is still commonly considered a form of touch transmitted through the surrounding water.

Taking inspiration from above deep-sea creatures, tactile-based sensing modality is an option for monitoring deep-sea manipulation in light-limited conditions. Embedded in a deep-sea gripper, tactile sensors are potential to provide feedback to contribute a non-destructive interaction during the collection of fragile specimens. As a typical electrical-based tactile sensor, resistive skins rely on the resistance changes of conductive liquid filled channel, which are less susceptible to electric noise, in comparison to capacitive modality. However, their suitability for deep-sea applications remains questionable due to potential issues such as thermal drift and freezing of internal liquid conductive materials. An optical waveguide-based tactile sensor is attached to a rigid gripper for object recognition and localization in a pressure chamber under 600 bars (or 592 atm)^82^ (Fig. 4b). This light modulation sensor was also integrated with soft robotic fingers to enable soft manipulation with touch sensing, showing a performance that is less susceptible to hydrostatic pressure^64^. Besides, a whisker-inspired piezoresistive sensor was designed for ultrasensitive underwater flow sensing and wake tracking^83^. These tactile sensing strategies have the potential to enable soft robots to perform intelligent tasks with feedback in the deep sea.

#### Contactless sensing

Certain deep-sea creatures (e.g., Benthobatis moresbyi) have evolved with special organs to emit weak electric field and electroreceptor arrays to detect their surrounding objects (Fig. 4c). By comparing the intensity of electric fields sensed by each electroreceptor, these creatures can achieve contactless perception and localization, a capability known as electrolocation^84^. With the same mechanism, a soft artificial electroreceptor consisting of an ionic conductive hydrogel-filled canal has been developed to detect the changes of electric field^85^. Similarly, the change in induced-voltage of the artificial receptors is correlated to the distance between the approaching object and the sensor. The integration of transmitter which emit electric field lines, and flexible electroreceptor array, will enable soft manipulator to locate and autonomously grasp objects^85^ (Fig. 4d). This technology can also enable soft swimmers to navigate and avoid obstacles in the deep sea.

In contrast, cetaceans use echolocation to pinpoint the location of objects at greater distances by emitting sound and detecting the reflected acoustic waves^86^ (Fig. 4e). Inspired by this concept, a bioinspired flexible acoustic transceiver, composed of piezoelectric electrodes attached to a flexible substrate, has been developed to achieve distance communication and echolocation. When functioning as a transmitter, the device transforms electric signal into a modulated acoustic wave. Simultaneously, as a receiver, it detects incoming acoustic waves and generates net charges on the electrodes, which can be converted into measurable electrical signals^52,87^ (Fig. 4f). The integration of this type of compact and flexible acoustic sensor can potentially enable soft robots to perform remote communication, sensing, and echolocation in harsh deep-sea conditions. To enhance the sensing distance and resolution ratio, future directions involve advancing materials with high piezoelectric coefficient and optimizing the distribution of the electrodes.

### Applications

#### Deep-sea soft manipulation

As a long-term hotspot of hadal exploration, deep-sea manipulation continually contributes to the work of biologists, geologists, and archeologist. Galloway et al. have made a significant breakthrough in ‘non-destructive deep-sea soft manipulation’, in which they present a soft gripper featuring ultra-gentle interaction when sampling delicate biological spices. Carried and powered by a ROV, this hydraulic soft gripper operates reliably at depths greater than 800 m and was able to collect soft coral at a depth of 100 m^61^. Vogt et al. have utilized 3D-printed soft hydraulic grippers to collect delicate deep-sea organisms at a depth of 2224 m. Real-time control from the ROV pilot enables delicate wrapping and manipulation of fragile deep-sea organisms^62^. Additionally, Stephen et al. have developed a universal jamming gripper by filling an enclosed elastic membrane with a mixture of water and glass beads. This design strategy is validated through a series of experiments in a hydrostatic pressure testing chamber and field tests in the deep sea (depth ≥ 1200 m)^63^.

In addition to grippers, soft robotic arms have been developed for deep-sea exploration. Phillips et al. have presented a multi-DOF soft manipulator that integrates bending, rotary, and grasping units, achieving dexterous movements and a delicate grasping motion. The deep-sea soft manipulator is actuated using the surrounding water and remotely controlled via a flexible, wearable sensor integrated into a compact glove. The feasibility of operation is validated under a hydrostatic pressure of 23 MPa, equivalent to a depth of 2300 m, as well as field-tested to demonstrate deep-sea sampling capabilities^60^. Also, soft robotic arms can be combined with locomotion for seabed operations. Modeling the locomotion strategies of benthic animals like octopuses, legged robots equipped with continuum arms have been developed to perform walking and manipulation on the largely unexplored seabed^88,89^. These achievements offer promising and effective solutions for delicate interaction with deep-sea specimens.

#### Deep-sea swimmers

Inspired by the unique body features of deep-sea snailfish, Li et al. have designed an untethered, DE-driven soft robotic fish that can operate at the seabed of the Mariana Trench. Without the need for any bulky metallic vessels, this soft robot can withstand an extreme pressure of 110 MPa. The swimming performance and pressure adaptability of this robot are rigorously validated through systematic experiments conducted in a pressure testing chamber. Notably, this soft robot exhibits free-swimming capabilities at a depth exceeding 3000 m. It is also field-tested and actuated reliably at a depth around 10,900 m in the deep sea^66^. This research paves the way for a new generation of deep-sea mobile explorers, highlighting the promising direction of bioinspired soft machines that can be used in extreme conditions.

### Challenges and outlooks

Early attempts in deep-sea exploration have showcased the impressive capabilities of soft robots, including delicate manipulation, untethered locomotion, and perceptions. The existing question lies in synergistically integrating those discussed powerful actuation, advanced sensing, self-energy regeneration, as well as envisioning practical applications for these bioinspired machines in real deep-sea exploration scenarios. More breakthroughs can be expected from the improved locomotor, sensing capabilities, embodied power regeneration, and sustainability for deep-sea soft machines. By addressing these challenges, researchers can unlock new possibilities and enhance the effectiveness of soft robots for deep-sea exploration.

#### Actuation

Due to their low output power, weak locomotor capabilities, and poor perception abilities, it is challenging for soft robots to effectively navigate and adapt to the unpredictable deep-sea environment. For instance, soft robotic fish often struggle to resist disturbances and move at slower speed compared to conventional deep-sea robots^67^. To address this issue, a primary effort is to develop soft active materials that offer deep-sea actuation featuring fast response, large deformation, high output force, and ease of untethered power. Electroactive actuators may stand as a better options to deliver these attributes compared to thermal responsive actuators.

Nevertheless, soft electroactive materials face a common challenge of increased elastic modulus at low temperature and high pressure, which hampers their actuation performance. As a potential solution, soft electrostatic hydraulics drive local redistribution of the internal liquid dielectric, enabling deformation of the soft structure without the need of an external pump^90–92^. One future direction is to develop liquid dielectric materials with a lower viscosity and increased dielectric constant, thereby offering large, quick, and low-energy actuation for self-contained deep-sea soft machines.

#### Sensing

From system design standpoint, the integration of sensing and actuation will enable deep-sea soft robots to better understand and react to their environment. For deep-sea sensing, the grand challenges lie in maintaining its precision, sensitivity, and accuracy, due to the change of material properties (e.g., elastic modulus, viscoelasticity) under low temperature and high pressure. For the ones that rely on deformations of a conductive material or liquid, the increase in pressure shifts the resting baseline of the sensor and alters the dynamic range^64,93^. Additionally, low temperatures cause bulk sensor materials to stiffen and the conductivity of conductors to change. Fundamentally, one approach is to use analytical models to calibrate and adjust the perception signals to account for the dynamic range. This calibration process can help maintain the accuracy and sensitivity of the sensors despite variations in material properties. Otherwise, integrating multiple sensor modalities (e.g., contactless modality, tactile) can increase the density and diversity of sensing capabilities, providing a more comprehensive understanding of the surrounding water. Ultimately, the integration of sensor skins into deep-sea soft robots offers the potential of real-time feedback in teleoperation, allowing operators to perceive the underwater environment through virtual reality and make informed decisions^94^. Furthermore, sensor skins enable high-level autonomous behaviors and task planning in the deep sea, facilitating the robot’s ability to adapt and perform complex tasks.

#### Embodied energy regeneration

The embodied energy storage that is mainly based on rechargeable battery, remains a major limitation for deep-sea robots in terms of long endurance to perform extensive operations. Previous attempts have been made to design energy-dense aquatic soft robots to ensure their long operation, by integrating flow shell battery into soft materials and structures instead of using separate battery packs^53,95^. Nevertheless, there is still a trade-off to be considered in the range of total mission time, size, weight, and mobility of the overall system. It is noteworthy that one species of white skate has been recently reported to brilliantly utilize hydrothermal heat for egg-case incubation, inspiring embodied energy harvesting/regeneration for deep-sea soft machines^23^. Driven by seafloor volcanism, hydrothermal vents are among the most spectacular features on the seabed and contain enormous thermal energy, which holds promising potential for exploitation^6–8^. In the future, untethered soft robots integrated with thermal electric skins-an energy harvesting system, may thrive near hydrothermal vents to derive energy, thus, unleashing the possibility of long-term and self-contained mission^96^. Future directions can focus on improving the flexibility and thermoelectric properties in the deep sea.

#### Manufacturing and sustainability

**Fig. 5:**
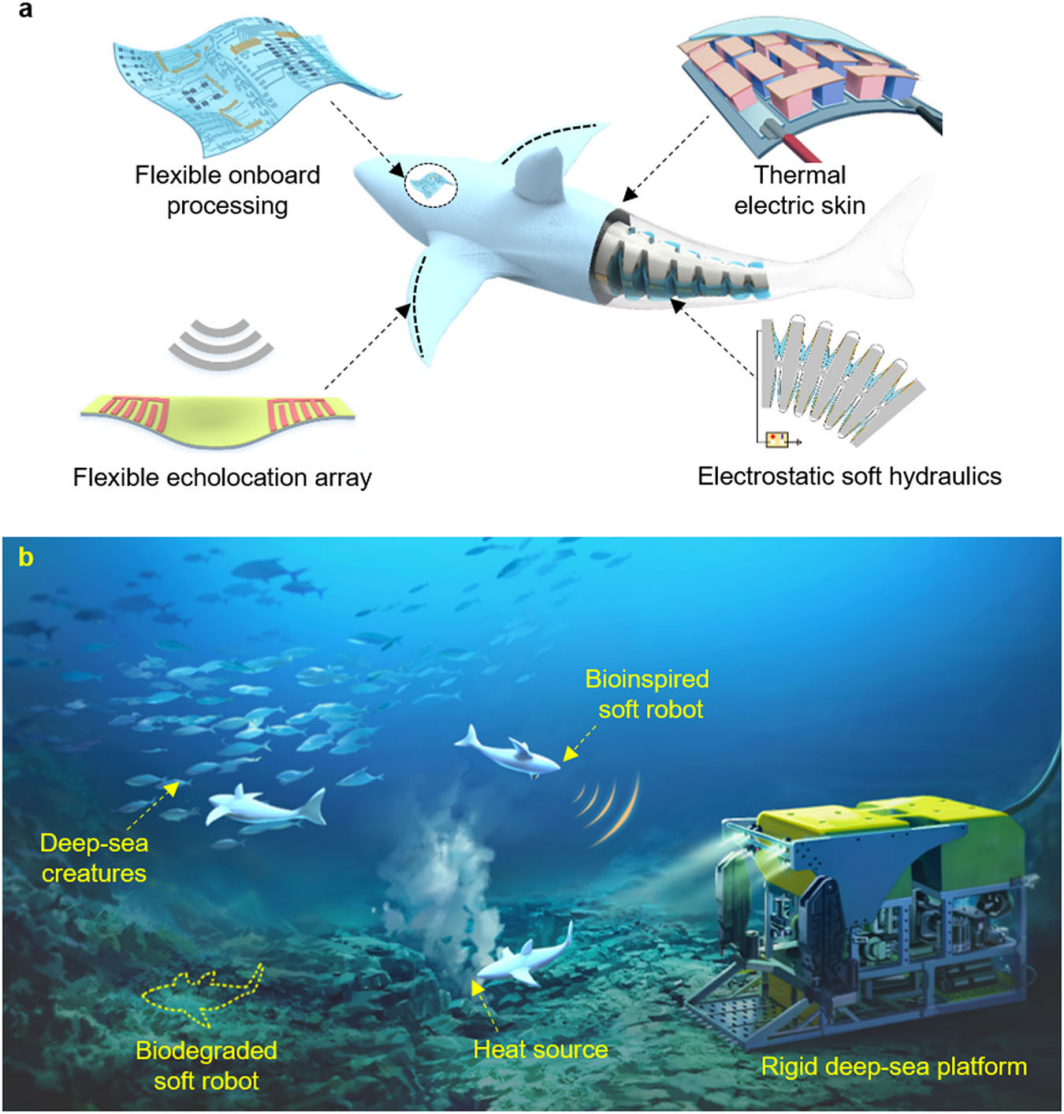
Perspective design and practical application of future deep-sea soft robots. **a** By integrating soft electrostatic hydraulic actuation, soft sensory arrays, embodied energy regeneration skins, and flexible onboard processing electronics, future deep-sea soft robots could enable long-endurance and more autonomous machine behaviors in deep-sea tasks^87^. Reproduced with permission under CC BY 4.0 license from ref. ^87^. **b** Bioinspired soft robots will enable scientists to perform in situ investigations of the deep-sea habitats, and to preserve the original deep-sea ecosystem.

For real-world applications, flexible circuits and electronics can be integrated into deep-sea soft robotic systems for power, sensing, and on-board processing^97^ (Fig. 5a). To achieve this, hierarchical and precise technologies for fabricating multi-material and multifunctional soft structures with pressure resilience should be advanced. At this point, soft robot fabrication should be considered in respect to diverse sustainability niches. It can begin with the selection of raw materials that are derived from natural resources or recycled materials, followed by establishing soft robot fabrication process with lower carbon footprint. At the end of their life cycle, the ideal building materials should either have self-healing properties to prolong lifespan or be capable of biodegrading into non-toxic constituents, ensuring no harm to the ecology and environment^98^. Developing deep-sea soft robot using self-healable materials faces challenges due to harsh conditions including extreme hydrostatic pressure, low temperatures, and corrosive seawater^99^. These factors can profoundly impact the performance and stability of self-healing materials. Therefore, it is crucial to develop materials that can withstand these conditions without compromising their healing capabilities. In the future, soft sustainable machines with integrated flexible onboard systems and artificial intelligence algorithms will offer more effective solutions for deep-sea tasks (Fig. 5a).

#### Future envisions

Despite the existing open questions in this area, we believe that bioinspired soft robot is a promising technology to initiate a completely different set of deep-sea missions in the future. Developments in actuation, sensing, energy, and manufacturing technologies will push the boundaries of soft machines for the deep ocean exploration, as well as other tasks in various harsh conditions. In the next decades, well-designed soft robots will safely navigate biotic community and coral reefs, as well as contribute to constructing seamless interaction between humanity and deep-sea creatures (Fig. 5b). Also, soft robots make it possible for researchers to perform in situ investigation of the deep-sea habitats, thus helping scientists to preserve the natural ecosystem and uncover more secrets in this endless frontier. We hope the design strategies, directions, and future opportunities discussed in this perspective will provide effective guidelines for deep-sea soft robotics and boost more breakthroughs in this field.
